# Disease of the Ear Causing Death

**Published:** 1847-06

**Authors:** 


*Disease of the ear causing Death.*—Dr. W. W. Merriman at a recent meeting of the Westminister Medical Society described a case of this description that had come under his care. A woman of scrofulous diathesis, aged twenty-six, was seized on January 26th with intense ear-ache, which extended over the whole right side of the head, without any alleviation from remedial means. Conciousness lasted complete until about thirty hours before her disease; but the mid-day preceding, she perfectly understood what Dr. Merriman said to her, put out her tongue, &c., and on the following morning she spoke to her friends; but except these occasions, she was more or less insensible; there were no appearances of convulsions or paralysis, but the jaws became fixed, so that she could scarcely open her mouth. She expired on the evening of February 18th.

On examination after death, a large abscess was found occupying the upper part of the right hemisphere, containing about two ounces of pus. This mostly escaped before removing the skull cap, being so close to the temporal bone at that point; here the dura mater was found dark colored, and separated from the bone, the diseased part being surrounded by a deposit of coagulable lymph on both sides of the dura mater. The mastoid cells of the temporal bone were examined by Mr. Toynbee, and found carious; they were full of a dark-colored pus, epithelial cells, &c. Several apertures existed in the lamina of bone forming the roof of these cells, which was extremely thin. The patient had had the measless when a child, suffered from discharge, at times offensive, from the ear ever since; had complained of headaches for the last year or sixteen months, and had during that time been very forgetful, and at times giddy; she had also fallen away very much. Dr. Merriman, taking these symptoms into account, as also the connexion so frequently found between the abscess within the cranium and carious bone of the base of the skull, was of opinion that the original desease of the ear had been communicated to the brain, causing abscess and death; and thence made some remarks upon the necessity of curing, if possible, the disease in the bones, to prevent the access ofthese more frightful disorders.—*Times.*

